# The Degree of Modulation of Beta Band Activity During Motor Planning Is Related to Trait Impulsivity

**DOI:** 10.3389/fnint.2019.00001

**Published:** 2019-01-17

**Authors:** Charidimos Tzagarakis, Andrew Thompson, Robert D. Rogers, Giuseppe Pellizzer

**Affiliations:** ^1^Department of Neuroscience, University of Minnesota, Minneapolis, MN, United States; ^2^Brain Sciences Center, Minneapolis VA Health Care System, Minneapolis, MN, United States; ^3^Department of Psychiatry, Warneford Hospital, University of Oxford, Oxford, United Kingdom; ^4^College of Biological Sciences, University of Minnesota, Minneapolis, MN, United States; ^5^School of Psychology, Bangor University, Bangor, United Kingdom; ^6^Department of Experimental Psychology, University of Oxford, Oxford, United Kingdom; ^7^Department of Neurology, University of Minnesota, Minneapolis, MN, United States

**Keywords:** impulsiveness, magnetoencephalography, beta band, motor cortex, motor planning

## Abstract

Impulsivity is a prominent personality trait, and a key modulating component of neurologic and psychiatric disorders. How impulsivity is related to the brain mechanisms associated with action planning is poorly understood. Here, we investigated the relation between impulsivity and the modulation of beta band oscillatory activity associated with action planning and execution. Given that beta power decreases during action planning and decreases further during action execution, we hypothesized that during planning the level of beta band power of more impulsive individuals would be closer to the level reached during execution than that of less impulsive individuals. This could explain the tendency to “jump the gun” (commission errors) in high impulsivity. To test this hypothesis, we recruited healthy volunteers (50 participants analyzed) and used the Barratt Impulsiveness Scale questionnaire to evaluate their impulsivity as high or low. We then recorded their brain neuromagnetic signals while they performed an instructed-delay task that induced different levels of action planning by varying the number of spatial cues, hence the uncertainty, about the location of the upcoming target. During the early cue period of the task, we found a posterior (source localized in the occipito-parietal areas) and a left fronto-central group of channels (source localized in the left sensorimotor areas) where beta power was modulated by number of cues, whereas during the late cue period only the left fronto-central group was modulated. We found that the decrease of relative beta band power during action planning in the left fronto-central group of channels was more pronounced in the high impulsivity group than in the low impulsivity group. In addition, we found that the beta band-mediated functional connectivity between the posterior and the left fronto-central groups of channels was weaker in the high impulsivity group than in the low impulsivity group during the early cue period. Furthermore, high impulsives made more commission and movement errors in the task than low impulsives. These results reveal neural mechanisms through which impulsivity affects action planning and open the way for further study of the role of beta band activity in impulsivity, especially in the context of disease and therapeutics.

## Introduction

Impulsivity has been defined as the predisposition to rapid, unplanned reactions to internal or external stimuli without regard for the consequences (Moeller et al., 2001). In both social and clinical settings, being impulsive is commonly described as “acting before thinking.” In other words, impulsivity evokes the notion of premature, unprepared action, as opposed to careful decision making. It is a prominent personality trait within the general population, and a key modulating component of neurologic and psychiatric disorders (Moeller et al., 2001), including Parkinson’s disease (Nombela et al., 2014), cluster B personality disorders (Wilson et al., 2007; Turner et al., 2017), bipolar affective disorder (Swann et al., 2001; Wilson et al., 2007), behavioral, drug and alcohol addictions (Rubio et al., 2008; Grant and Chamberlain, 2014; Mei et al., 2015) and attention deficit hyperactivity disorder (ADHD; Sharma and Couture, 2014).

Impulsivity has progressively been established as a multi-dimensional construct (Evenden, 1999; Robbins et al., 2012) that has been measured using behavioral tests as well as self-report questionnaires (Evenden, 1999; Tzagarakis et al., 2013). In addition to risk-taking and the pursuit of immediate as opposed to long term reward, often referred to as “delay discounting” (Ciccarelli et al., 2016), inhibitory control dysfunction is thought to be a key characteristic of excessive impulsivity (Enticott et al., 2006). Indeed, several studies have shown that self-reported measures of the impulsivity personality trait are associated with “commission errors” in stop-signal, go/no-go, Stroop, and other tasks that require the inhibition of a preponderant response (Enticott et al., 2006; Lansbergen et al., 2007; Saunders et al., 2008; Burle et al., 2016). However, there is also evidence that impulsivity affects action planning in general, that is, not only when a preponderant response ought to be inhibited, both in health (Tzagarakis et al., 2013; Rossi et al., 2018) and in disease (Clark et al., 2006; Kertzman et al., 2010; Mortensen et al., 2016).

How impulsivity modulates the brain mechanisms associated with action planning is poorly understood. Here, we used known phenomena underlying the electrophysiology of action planning to investigate this question. It has been known for a long time that the modulation of motor system beta band (∼15–30 Hz) oscillations plays a key role in action planning and execution (Jasper and Penfield, 1949; Pfurtscheller, 1981). In particular, action planning is associated with a decrease in beta power (also known as “beta desynchronization” in the literature) which decreases even further during action execution (Tzagarakis et al., 2010; Grent-’t-Jong et al., 2014; Heinrichs-Graham and Wilson, 2016). In other words, the decrease in beta band power during action planning reflects the change of the motor system from a resting state to a state closer to the one associated with action execution. For this reason, we hypothesized that the decrease in power of beta band oscillations is more pronounced in more impulsive individuals than in low impulsive individuals. If the state of the motor system of impulsive individuals during action planning gets closer to the state associated with action execution, then that would provide a physiological explanation for the greater occurrence of commission errors in impulsive individuals.

In addition, there is evidence that high impulsive individuals have reduced functional connectivity between brain areas associated with motor planning and those associated with spatial attention (Shannon et al., 2011; Vaidya and Gordon, 2013), and there is also growing evidence for a role of beta oscillations in perception at occipito-parietal areas (Kloosterman et al., 2015; Meindertsma et al., 2017). For those reasons, we hypothesized that beta oscillatory activity within the action planning occipito-frontal network is modulated by impulsivity. More specifically, since perceptual information plays a lesser role in decision making when impulsivity is high (Clark et al., 2006), we expected that beta band functional connectivity between regions with a motor and a perceptual role would be diminished in individuals with high as opposed to low impulsivity.

To test these hypotheses, we recruited neurologically and psychiatrically healthy individuals and used the Barratt Impulsiveness Scale questionnaire (Patton et al., 1995) to evaluate their trait impulsivity. The Barratt scale is a widely used self-report measure of trait impulsivity in studies with healthy and pathological populations (Stanford et al., 2009). We then recorded the participants’ brain neuromagnetic signals while they performed an instructed-delay task that induces different levels of motor planning by varying the uncertainty about the location of the upcoming target (Pellizzer and Hedges, 2003; Tzagarakis et al., 2010). Thus, in contrast to tasks designed to test the inhibition of a planned response (e.g., stop-signal, and go/no-go tasks), this task is designed to test action planning by manipulating how much planning can be done in advance (Pellizzer and Hedges, 2003; Tzagarakis et al., 2010). Accordingly, the reaction time in this task is affected by the early stages of pre-movement motor planning and inhibition, rather than by late stage inhibition such as the one seen in, for example, the stop-signal task (Logan et al., 1997). Finally, the instructed-delay in the task helps separating the perceptual and motor planning processes.

We have found previously that the decrease of relative beta power over motor areas during motor planning is inversely related to the degree of uncertainty, that is, the greater the uncertainty, the less beta power decreases during motor planning. We expected that more impulsive individuals would show a more pronounced decrease of beta power during motor planning, and that they would initiate a response before the go signal (i.e., commission error) more often than low impulsive individuals. We also posited that motor areas and areas associated with spatial attention showing beta band modulation from task conditions would have diminished functional connectivity in high impulsive individuals than low impulsive ones. The results supported these hypotheses.

## Materials and Methods

### Participants

Fifty-five healthy right-handed volunteers (30 women, 25 men) were recruited through posters placed on University grounds and advertisements in the local press. Exclusion criteria were age over 40 years, self-reported left handedness, active neurological or mental illness (including borderline personality disorder), active alcohol/drug misuse, and taking medication affecting the central nervous system. Participants were screened for mental illness and substance misuse using the Mini International Neuropsychiatric Interview (Sheehan et al., 1998), as well as the items of the Structured Clinical Interview for DSM-IV axis II (SCID-II; First et al., 1997) specific to borderline personality disorder. The study protocol was approved by the University of Oxford Research Ethics Committee. All participants provided written informed consent before the experiment in accordance with the Declaration of Helsinki. The data of five participants were discarded from the analyses due to technical problems during data acquisition. The dataset analyzed thus included the data of fifty participants. Age and sex distribution of the participants are given in Table 1.

**Table 1:** Age and Barratt Impulsiveness Scale (BIS) scores of the participants.

	Low impulsives			High impulsives			Low + High		
	N	Age, years	Total BIS score	N	Age, years	Total BIS score	N	Age, years	Total BIS score
**Women**	15	24.3 (4.1)	50.7 (8.0)	13	22.9 (3.7)	75.8 (9.1)	28	23.6 (3.9)	62.4 (15.3)
**Men**	9	23.0 (2.3)	50.7 (5.0)	13	22.9 (5.8)	72.8 (7.1)	22	23.0 (4.6)	63.7 (12.7)
**Women + Men**	24	23.8 (3.5)	50.7 (6.9)	26	22.9 (4.8)	74.3 (8.2)	50	23.3 (4.2)	63.0 (14.1)

*Mean (SD)*

### Impulsivity

**FIGURE 1:**
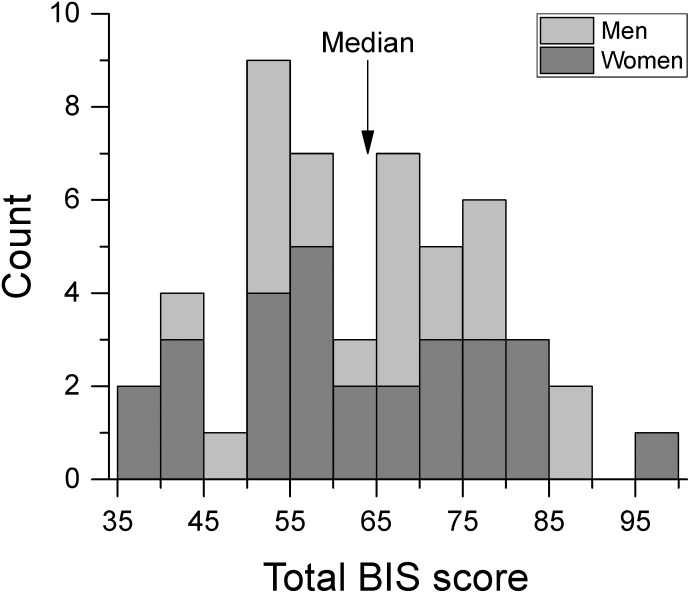
Frequency histogram of total BIS score by sex group. We separated participants into low and high impulsivity groups at the median BIS score.

Participants completed the Barratt Impulsiveness Scale version 11 (BIS-11; Patton et al., 1995). The questionnaire is composed of 30 items regarding impulsive and non-impulsive behavior rated on a 4-point Likert-type scale. Total BIS-11 scores can vary between 30 and 120 with higher scores indicating greater impulsivity. Figure 1 shows the distribution of BIS-11 scores for men and women. These scores were not significantly different from those of healthy population normative data [Spinella, 2007; *t*-test(748) = 0.773, *p* = 0.440]. For the analyses of the effect of impulsivity, subjects were split into low and high impulsivity groups relative to the median of the total BIS-11 score (median BIS-11 score = 64) which gave *N* = 24 in the low impulsivity group, and *N* = 26 in the high impulsivity group. Table 1 shows the mean BIS-11 score and age of participants for the total dataset as well as by sex and by impulsivity group. We found no significant association between sex and impulsivity group [χ^2^(1) = 0.791, *p* = 0.374]. In addition, age was not significantly different across sex group [*F*(1,46) = 0.262, *p* = 0.611], impulsivity group [*F*(1,46) = 0.330, *p* = 0.569], or their interaction [*F*(1,46) = 0.262, *p* = 0.611].

### Task

**FIGURE 2:**
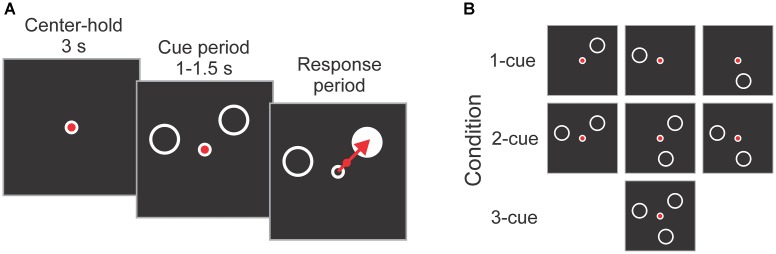
**(A)** Schematic diagram of the task. Each trial started with a 3 s center-hold period, followed by a 1–1.5 s cue period in which 1, 2, or 3 cues (empty circles) indicated the possible locations of the upcoming target. At the end of the cue period, one of the cues became the target (filled circle), and the subject reached it as rapidly and accurately as possible using a joystick-controlled cursor. **(B)** Cue configurations used in the experiment. They consisted of all possible combinations of 1, 2, and 3 cues at 3 locations (i.e., 45°, 165°, and 285°). The targets were equally represented at all 3 locations, and the order of the trials was pseudo-randomly mixed.

The participants performed an instructed-delay reaching task, as used in previous work (Pellizzer and Hedges, 2003; Tzagarakis et al., 2010, 2013). A diagram illustrating the task is shown in Figure 2A. Each trial started with a 3 s center-hold period during which a joystick-controlled cursor had to be maintained within a small circle presented in the center of the screen. The center-hold period was followed by a cue period that varied randomly and uniformly from 1 to 1.5 s during which 1, 2 or 3 peripheral cues (open circles) were presented. The cues indicated the possible locations of the upcoming target. The joystick-controlled cursor needed to be maintained in the center of the screen during the cue period. At the end of the cue period one of the cues was replaced by a target (filled circle) which had to be intercepted by moving the cursor from the center onto it as rapidly and precisely as possible. In addition, participants were instructed to visually fixate the central circle from the center-hold period to target onset. Reaction time (RT) was defined as the time between target onset and the cursor exiting the central circle. Movement time (MT) was the time from the cursor exiting the central circle to the time it hit the target.

The 1, 2 or 3 cues presented during the cue period were located at any combination of three possible locations: 45 deg, 165 deg and 285 deg (angles defined on the unit circle), which gives a total of 7 cue combinations (i.e., 3 × 1-cue, 3 × 2-cue, and 1 × 3-cue; see Figure 2B). Each subject completed 84 trials per number of cues condition equally balanced across target directions. This resulted in 252 trials which were presented in a pseudo-random order. The trials were divided into 2 blocks of 126 trials with a brief rest period between blocks.

Initiating a response before the target appeared or in less than a minimum RT set at 100 ms, resulted in an “early response error” (commission error). A successful interception of the target required that the cursor trajectory remained within a straight path from the center to the target (the width of the path had the same width as the target), and that the cursor stayed within the target for at least 100 ms. If either of these two conditions was not met the trial was considered as a “movement error.” Feedback about errors and correct responses was signaled to the participant using different beep tones. Unsuccessful trials were reinserted in the remaining pool of trials until all trials were completed successfully.

Participants were given a brief period of practice with the task before data acquisition. The task was implemented using custom-made software written in Visual Basic 6 (Microsoft, Redmond WA, United States) and running on a Windows personal computer.

### MEG Data Acquisition

Data acquisition was performed at the Oxford Centre for Human Brain Activity using a VectorView-306 MEG scanner (Elekta Neuromag Oy, Helsinki, Finland) with 306 sensors consisting in 204 planar gradiometers and 102 magnetometers. Each element of the detector array is composed of a magnetometer and two orthogonal planar gradiometers. Data from all sensors were used for pre-processing the data, but only the data from the gradiometers were used in the main analyses. Participants performed the task while sitting comfortably inside a magnetically shielded room where brain neuromagnetic activity was recorded at a sampling frequency of 1,000 Hz with a high-pass filter of 0.03 Hz and a low-pass filter of 330 Hz. The joystick-controlled cursor and task stimuli were back-projected on a screen located at a distance of 1.25 m in front of the participant. The distance between the center of the screen and the outer edge of the cue and target stimuli subtended 2.3 degrees of visual angle. Manual responses were recorded using a custom-made non-magnetic joystick controlled with the right hand. The joystick was placed on a flat surface in front of the subject. Synchronization between task events and MEG data was assured using a photodetector placed on the projection screen with output in one of the MEG auxiliary channels. Eye blinks were recorded using a pair of surface electrodes positioned above and below the eye. The head shape and three fiducial points (viz, nasion, left and right pre-auricular points) were recorded using a 3-D digitizing stylus (Polhemus, Colchester, VT, United States).

### MEG Data Pre-processing

MEG data were de-noised using the spatio-temporal extension to Maxwell filtering (MaxST) (Taulu et al., 2005; Taulu and Simola, 2006) implemented in MaxFilter version 2.1.15 (Elekta Neuromag Oy, Helsinki, Finland). The rest of the pre-processing steps were performed using Fieldtrip (Oostenveld et al., 2011). First, cardiac artifacts were removed from the data using Independent Component Analysis (ICA). Briefly, for every subject and block of trials, the gradiometer and magnetometer data were subjected to ICA decomposition. The resulting components were visually inspected to identify those corresponding to electrocardiogram signals. These components were then removed and the remaining ones were back-projected to channel space. All subsequent data pre-processing and analyses were then carried out using only data from the 204 gradiometers. Trials contaminated by muscle artifacts, eye blinks and electronic artifacts (SQUID jumps) were discarded using an automatic threshold-based approach. As a consequence of this thresholding, 7% of trials were removed from the analyses. The data were detrended, low-pass filtered (anti-aliasing 125 Hz low-pass Finite Impulse Response bidirectional filter), and then down-sampled to 250 Hz to facilitate further processing.

### MEG Data Processing and Statistical Analyses

MEG data were processed and analyzed using the FieldTrip (Oostenveld et al., 2011) MATLAB toolbox in combination with custom-made MATLAB (Mathworks Inc., Natick, MA, United States) scripts. Additional statistical analyses were performed using the Microsoft R Open implementation of R 3.4.2 (R Core Team, 2017).

### MEG Data Per Channel

For each channel, time-frequency signal decomposition was computed at the trial level from 2 to 40 Hz at 1 Hz increments using Morlet wavelets of 7 cycles, and at 20 ms steps. After examination of normalized channel time-frequency maps averaged across all channels and trials to confirm the expected task-related beta desynchronization, the beta band was defined using a central frequency of 22 Hz and ± 7 Hz width, which is consistent with previous studies (Tzagarakis et al., 2010, 2015; Grent-’t-Jong et al., 2014; Heinrichs-Graham and Wilson, 2016). We extracted cue-aligned and target-aligned data of beta band power per trial for each subject and channel. For each number of cues condition, time-frequency bins within the frequency band of interest were summed and normalized relative to baseline (-0.6 to -0.1 s before cue onset) and then log transformed. Statistical analyses were performed on relative power averaged over epochs of interest: (1) early cue period (0–0.5 s after cue onset); (2) late cue-period (0.5–1 s after cue onset); and (3) response period (0.5 s window from 0.1 s before to 0.4 s after the grand mean RT; i.e., 0.3 to 0.8 s after target onset).

### Channels Modulated by Number of Cues Conditions

Before examining the effect of impulsivity, we sought channels for which the power in the beta band was modulated by the number of cues conditions during epochs of interest. To this end, we calculated for each channel the *t*-value of the dependent samples regression of relative power against number of cues conditions (1, 2, and 3 cues). Then, we used a cluster-based non-parametric method to determine the statistical significance of power modulation by number of cues conditions (Maris and Oostenveld, 2007). The first step for the cluster-based statistic is the selection of the threshold that determines inclusion in a cluster. That choice affects the test sensitivity but does not affect the statistical validity of the test (Maris and Oostenveld, 2007). We set the cluster membership *t*-value threshold in the following way. The cluster-based statistics were computed repeatedly using a regularly increasing threshold starting from a *t*-value of 2.0 (2-tailed *p* = 0.05 with df = 49) up to a *t*-value of 10.0 (2-tailed *p* << 0.001 with df = 49) with *t*-value increments of 0.2. We generated plots of the number of selected channels and of the cluster-summed *t*-values versus the threshold *t*-value. Then we chose the threshold by identifying the inflection point in the plots. Separate thresholds were selected for positive and negative modulations of power with number of cues. The statistical significance of the clusters was determined as follows. For each subject, relative power data were randomly assigned to each number of cue condition before calculating the cluster-summed *t*-values for the dependent samples regression. This procedure was repeated 10,000 times to create a distribution of the cluster statistic under the null hypothesis of no relation between relative power and number of cues condition. The tests for cluster significance were done for the three epochs of interest (i.e., early cue, late cue, and response periods). Bonferroni correction for multiple comparisons was thus used to control the family-wise error rate at 0.05 (Shaffer, 1995) which resulted in a 2-tailed significance threshold of *p* = 0.017. Channel groups were formed by all the channel clusters with significant summed *t*-value of the same sign. Finally, we computed the time series of relative power (expressed in dB) for each group of channels that had significant modulation of power with number of cue conditions during epochs of interest.

### Source Level Analysis

Our dataset lacked individual anatomical MRI data. However, we performed source localization analyses using individual head shape data in order to localize task-related anatomy. Three subjects were excluded from this analysis due to the lack of useable head shape data. Specifically, we performed *post hoc* analyses to localize relative power modulation during epochs that had a significant channel level effect of number of cues on the modulation of beta band relative power. For each subject, a single-shell model of the brain surface (Nolte, 2003) was created based on the digitized individual head shape and the Montreal Neurological Institute (MNI) magnetic resonance imaging brain template (Fonov et al., 2009). The brain volume was divided into a regular MNI normalized 8 mm voxel grid, and a lead field matrix was computed for each grid location. The localization of neural sources was performed using the Dynamic Imaging of Coherent Sources (DICS) beamformer (Gross et al., 2001). DICS was computed using the beta band multi-taper cross-spectral density matrix from the trials of all number of cues conditions to obtain a common spatial filter, and by using singular value decomposition with a regularization parameter of 10 %. Source power in the epochs of interest was computed for each number of cues condition using the common spatial filter, normalized to baseline and log-transformed. The source level statistical analysis was done as for the channel level analysis, in that we computed the dependent samples regression of relative power across number of cues conditions, and the cluster membership threshold and significance of clusters of voxels were computed using the method described above.

### Effect of Impulsivity

The effect of impulsivity was assessed for each group of channels modulated by number of cues conditions. Mixed effect models were tested using the R library nlme (Pinheiro et al., 2017) with relative power as dependent variable; sex, number of cues condition, and impulsivity group as fixed factors; and subject as random factor. The within-subject correlation between measures was modeled using the compound symmetry covariance structure. We tested several fixed effect models from a full factorial model to simplified models and selected the one with the lowest Akaike information criterion (AIC; Akaike, 1973). This model included sex, number of cues condition, and impulsivity group main effects, and the number of cues condition x impulsivity group interaction. Wald chi-square tests for type III sum of squares ANOVAs were computed for this model. The effect of impulsivity was tested for each epoch and group of channels modulated by task condition (see above), and for that reason we used Bonferroni correction for multiple comparisons to adjust the significance threshold (Shaffer, 1995). In addition, if there was a significant effect of impulsivity on a group of channels, we performed additional analyses of baseline data for those channels using log-transformed non-normalized beta band power as the dependent variable and a reduced model comprising of main effects for sex and impulsivity group only.

### Channel Groups Functional Connectivity

The analysis of channels modulated by the task conditions during the early delay period revealed the presence of two groups of channels with different localization and reversed effects of task conditions. For this reason, we analyzed whether impulsivity affected the interaction between these two groups of channels during the task. To this end, we computed the time-varying average amplitude correlation coefficient between channel groups. The amplitude correlation metric has good test-retest reliability in beta band (Garcés et al., 2016) and is robust to the temporal jitter at relatively high frequencies such as beta compared to phase-based metrics (Cohen, 2014). Briefly, for every subject, broadband signal from each trial and channel in the two channel groups was bandpass filtered within the 15-29 Hz frequency range with a two-pass band-pass Finite Impulse Response filter. The amplitude of the resulting signal time series was calculated as the modulus of its analytical signal after Hilbert transform and then log transformed. This resulted in the creation of a high-resolution time series of log-transformed beta band amplitude for each trial and channel. For each time point of each trial the Pearson correlation coefficient of data within a 180 ms time window centered on it was calculated between all pairs of channels across the two groups. The resulting correlation values *r* were then transformed using Fisher transform *z* = arctanh*(r)* to stabilize their variance (Cohen, 2014) and averaged across trials and channel pairs to create a time series of average Fisher z-transformed amplitude correlation between channel groups. This metric provided a connectivity measure of covariation of beta power between channel groups. The effect of sex, number of cues condition, and impulsivity group during the early delay period was evaluated with a mixed effect model identical to the one used to evaluate relative beta power.

### Analysis of Behavioral Performance

Four behavioral measures (i.e., mean reaction time, mean movement time, counts of early response errors, and counts of movement errors) were analyzed using generalized linear mixed effects models. These models were defined using sex, number of cues, impulsivity group, and number of cues x impulsivity group interaction as fixed effects, and subject as a random factor. Appropriate distributions and link functions were selected after examination of AIC values and plots of residuals against fitted values. Mean reaction time and movement time were modeled using the gamma distribution, whereas error counts were modeled using the negative binomial distribution. A log link function was used in all cases. The models were fitted using the R package lme4 (Bates et al., 2015). Wald statistics were computed for ANOVAs with type III sums of squares.

In addition, we performed a *post hoc* analysis to explore further the effect of impulsivity and beta desynchronization on reaction time (Tzagarakis et al., 2010). To this end, for each participant we computed the change of reaction time from the mean as the dependent variable and used the relative beta power during the late cue period as covariate, and impulsivity group as factor. The analysis was performed using the same approach as described above. The Normal distribution with the identity link function was used to model the change in reaction time.

## Results

### Channels With Beta Band Power Modulated by Number of Cue Conditions

First, we computed the dependent samples regression between relative beta power and number of cues to search for groups of channels for which the power of the beta band was modulated by cue condition during the early cue period, late cue period, or response period. The cluster-based permutation test indicated the presence of significant modulation (Bonferroni corrected significance level at *p* < 0.017) during the early cue period, and the late cue period, but not during the response period.

#### Early Cue Period

**FIGURE 3:**
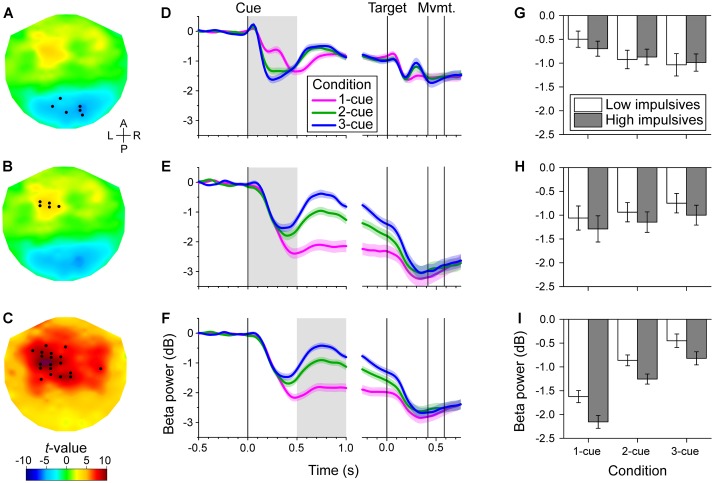
Channel-level analysis of beta band power during the task. We found that the power was significantly correlated with the number of cues during the early cue period (0–0.5 s after cue onset) and during the late cue period (0.5–1 s after cue onset), but not during the response period. **(A–C)**. Topographic maps of the *t*-values for the dependent samples regression between relative beta power and number of cues during the early cue period **(A,B)**, and during the late cue period **(C)**. The *t*-values were interpolated between channels using a thin-plate spline for graphic purposes. The black dots identify the channels that were selected by the cluster-based analysis. The early cue period was characterized by two groups of channels, a posterior group with a slight preponderance to the right, of negative correlation with number of cues **(A)**, and a left fronto-central group of positive correlation with number of cues **(B)**. In contrast, the late cue period was characterized by a predominantly left fronto-central group of channels of positive correlation with number of cues **(C)**. The anterior-posterior (A–P) and left-right (L–R) axes are indicated in **A**. **(D–F)**. Cue and target aligned time series of mean beta band relative power across cue conditions for the channels identified in the same row on the left topographic maps. The shaded areas along the curves indicate the standard error of the mean (*N* = 50 participants). The gray rectangles identify the epoch of interest. Vertical lines indicate, from left to right, cue onset, target onset, grand mean of movement onset (RT) and offset (MT). The decrease in beta band power was greater the greater the number of cues for the posterior channels during the early cue period **(D)**. In contrast, the decrease of beta band power was smaller the greater the number of cues for the left fronto-central group during the early cue period **(E)**, and late cue period **(F)**. **(G–I).** Bar graphs of relative beta band power across cue condition and impulsivity group for the channels and epoch of interest identified on the same row. Error bars represent the standard error of the mean (Low impulsivity *N* = 24; High impulsivity *N* = 26). The decrease of beta band power of the left fronto-central channels during the late cue period was significantly greater for the high impulsivity group than for the low impulsivity group **(I)**. The same tendency was observed for the left fronto-central channels during the early cue period **(H)**, although that was not significant. There was a number of cues x impulsivity group interaction for the posterior channels during the early cue period (see text), although this did not survive Bonferroni correction **(G)**.

During the initial part of the cue period, we found two distinct areas in which the power of the beta band was correlated with number of cues: a posterior area (bilateral but with a slight preponderance to the right) and a left fronto-central area (Figures 3A,B). Noticeably, the change of power was modulated in opposite directions in these two areas as indicated by the opposite signs of the *t*-values. In the posterior area, beta power was more strongly reduced the greater the number of cues (cluster-based statistic, *p* < 0.017). In contrast, in the left fronto-central area beta power was more strongly reduced the smaller the number of cues (cluster-based statistic, *p* < 0.017). The time series of relative beta power in the different cue conditions for these two channel groups is shown in Figures 3D,E. These time series illustrate the dynamic change in beta power during the task and the effect of number of cues during the early cue period (light gray background) which went in opposite directions in the two channel groups.

#### Late Cue Period

During the late part of the cue period, we found significant correlation between the beta band power and number of cues in channels that were predominantly located in the left fronto-central area (Figure 3C). In these channels, the power of the beta band was more strongly reduced the smaller the number of cues (cluster-based statistic, *p* < 0.017). The time series of relative beta power in the different cue conditions for this channel group are shown in Figure 3F. The time series show the differential effect of number of cues during the late cue period (light gray background).

#### Response Period

During the response period, we found no significant correlation between beta band and number of cues (cluster-based statistic, *p* > 0.017). The target-aligned time series in Figures 3D–F show that the power of the beta band decreased further during the response period relative to the cue period and reached a common level for all cue conditions.

### Source Analysis of Beta Band Power Modulated by Number of Cues Conditions

**FIGURE 4:**
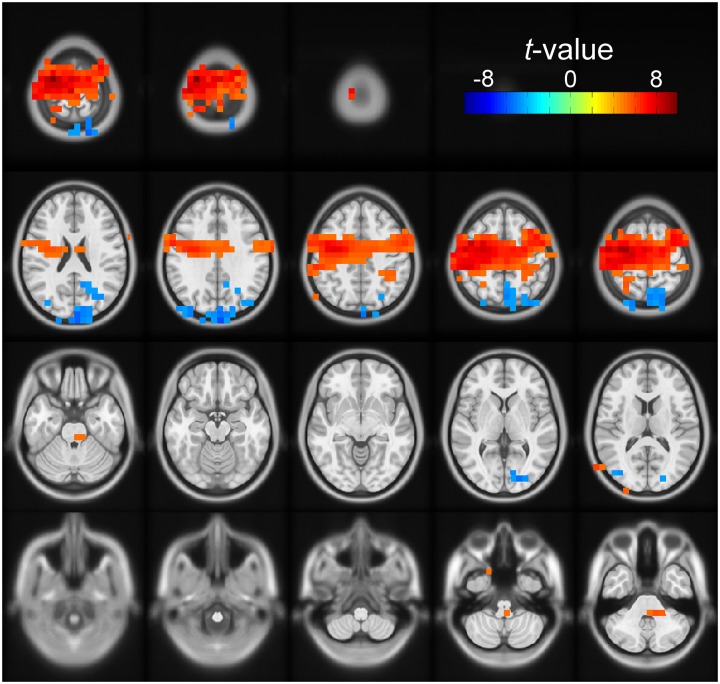
Source space solution rendered on an MNI brain template of the dependent samples correlation between relative beta band power and number of cues. Orientation is as for the channel plots in Figure 3. The *t*-values of the correlation are color-coded as indicated by the scale on the top right. The figure shows the clusters of voxels with significant negative (blue) modulation during the early cue period (0–0.5 after cue onset). The negative *t*-values indicate that relative beta band power decreased further as the number of cues increased. These voxels were localized bilaterally but with a right preponderance, mostly on the right superior parietal cortex, the right superior occipital cortex, the right precuneus, and the left cuneus. The figure also identifies the clusters of voxels with positive modulation by number of cues during the late cue period (0.5–1 s after cue onset). The positive *t*-values indicate that relative beta band power decreased less as the number of cues increased. These voxels were localized bilaterally but predominantly on the left sensorimotor area.

For both the early and late cue period, the channel-level analysis rejected the null hypothesis of no correlation between beta power and number of cues condition. To define better the anatomical areas involved in these effects, we performed a *post hoc* source analysis using a DICS beamformer. During the early cue period, we found significant modulation of beta band power by number of cues in the posterior occipito-parietal cortex. Voxels were localized bilaterally, but mostly in the right superior parietal cortex, the right superior occipital cortex, the right precuneus, and the left cuneus (cluster-based statistic, *p* < 0.05). During the late cue period, we found significant modulation of beta band power by number of cues mainly in sensorimotor areas. Voxels were localized bilaterally but predominantly in the left precentral, and left postcentral gyri, as well as in the left superior frontal gyrus, and left supplementary motor area (cluster-based statistic, *p* < 0.05). Figure 4 shows a rendering of the cortical sources identified for the two periods analyzed.

### Effect of Impulsivity on the Modulation of the Beta Band

For each group of channels identified in the previous analysis, we tested whether impulsivity affected the modulation of power in the beta band across cue conditions. As three channel groups were examined (i.e., two from the early cue period, and one from the late cue period), the family-wise error rate of 0.05 was controlled using Bonferroni correction to adjust the significance level at *p* < 0.017. We found significant modulation of beta power by impulsivity in the left fronto-central channels during the late cue period only.

#### Early Cue Period, Posterior Group of Channels

Figure 3G shows bar graphs of relative beta power for the posterior group of channels across cue condition and impulsivity group. As expected, there was a significant effect of number of cues [χ^2^(2) = 65.584, *p* < 0.001], with more pronounced decrease in beta power as number of cues increased [linear contrast: *t*(96) = -7.655, *p* < 0.001]. However, we found no significant effect of sex [χ^2^(1) = 0.003, *p* = 0.957], impulsivity group [χ^2^(1) = 0.046, *p* = 0.830], or cue condition x impulsivity group interaction [χ^2^(2) = 7.387, *p* = 0.025, which is not significant at the Bonferroni-corrected significance threshold].

#### Early Cue Period, Left Fronto-Central Group of Channels

Figure 3H shows bar graphs of relative beta power for the anterior group of channels across cue condition and impulsivity group. As expected there was a main effect of number of cues condition [χ^2^(2) = 25.068, *p* < 0.001], with less pronounced decrease in beta power as number of cues increased [linear contrast: *t*(96) = 4.877, *p* < 0.001]. However, we found no significant effect of sex [χ^2^(1) = 2.176, *p* = 0.140], impulsivity group [χ^2^(1) = 2.465, *p* = 0.116], or cue condition x impulsivity group interaction [χ^2^(2) = 0.130, *p* = 0.937].

#### Late Cue Period, Left Fronto-Central Group of Channels

**FIGURE 5:**
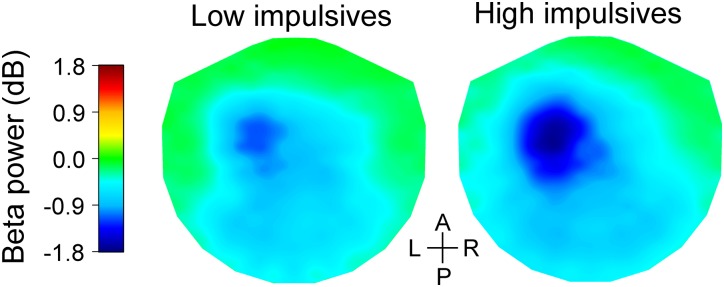
Topographic maps of relative change in beta band power during the late cue period of the task for the low (left) and high (right) impulsivity groups. The beta band power data were averaged across cue conditions. The data were interpolated between channels using a thin-plate spline. Note that the high impulsivity group had a greater decrease in beta band power over the left fronto-central area than the low impulsivity group. The anterior-posterior (A-P) and left-right (L-R) axes are shown in the middle.

Figure 3I shows bar graphs of relative beta power for the anterior group of channels across cue condition and impulsivity group. As expected, we found that there was a main effect of number of cues [χ^2^(2) = 191.063, *p* < 0.001], with less pronounced decrease of beta power as number of cues increased [linear contrast: *t*(96) = 13.265, *p* < 0.001]. We found that beta power in that group of channels was not significantly affected by sex [χ^2^(1) = 3.291, *p* = 0.070]. However, there was a significant effect of impulsivity group [χ^2^(1) = 7.623, *p* = 0.006], with more pronounced decrease in beta power for the high impulsivity group than for the low impulsivity group. We found no significant effect of the interaction cue condition x impulsivity group [χ^2^(2) = 0.886, *p* = 0.642]. The effect of impulsivity on beta band power during the late cue period can also be appreciated in Figure 5 which illustrates the topographic maps of relative beta band power for the low and high impulsivity groups. The figure shows that beta band power during the late cue period decreased noticeably more for the high impulsivity group than for the low impulsivity group.

In addition, given the significant effect of impulsivity on this group of channels during the task, we checked whether there was a difference in beta band power during the baseline period between the two impulsivity groups. To this end, we analyzed the non-normalized log-transformed power values of the baseline period and found no significant effect of impulsivity [χ^2^(1) = 0.265, *p* = 0.607], or of sex [χ^2^(1) = 0.725, *p* = 0.395].

### Functional Connectivity Between Posterior and Left Fronto-Central Beta Oscillations During the Early Cue Period

**FIGURE 6:**
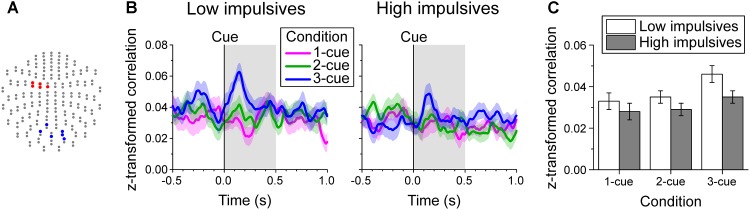
Beta band-mediated functional connectivity during the early cue period. **(A)** Channel groups (blue and red) with significant modulation of beta band power by cue condition during the early cue period of the task identified on the two-dimensional projection of the 204-gradiometer array (same as in Figures 3A,B). The functional connectivity between these two groups of channels was evaluated by computing for each trial the time-varying Pearson correlation coefficient of log-transformed beta band amplitude within a 180 ms window between each pair of channels from the posterior and left fronto-central groups. The Fisher’s z-transformed correlation coefficients were then averaged for each time point. **(B)** Cue aligned time series of average z-transformed correlation coefficient of beta amplitude between channel groups indicated in **A**, across cue condition and impulsivity group. The shaded bands show the standard error of the mean (Low impulsivity *N* = 24; High impulsivity *N* = 26). **(C)** Average z-transformed correlation of beta amplitude during the early cue period, across cue condition and impulsivity group. Error bars indicate the standard error of the mean. The correlation increased as number of cues increased and was smaller for the high impulsivity group than for the low impulsivity group across all cue conditions.

Since we found two groups of channels (posterior and left fronto-central, Figures 3A,B, 6A) with significant modulation of beta band power as a function of number of cues during the early cue period of the task, we investigated whether impulsivity modulated their interaction. To this end, we evaluated the relation of beta band oscillations between these two groups of channels by computing the Pearson correlation coefficient of their amplitude within a sliding time window. The time-varying average Fisher’s z-transformed Pearson correlation coefficient per cue condition, and impulsivity group is plotted in Figure 6B.

Figure 6C shows the average z-transformed correlation during the early cue period across number of cues and impulsivity group. The analysis showed that there was a significant effect of number of cues condition [χ^2^(2) = 10.216, *p* = 0.006; linear contrast: *t*(96) = 2.898, *p* = 0.005], due to a greater increase in correlation with number of cues after cue onset. We found no significant effect of sex [χ^2^(1) = 1.140, *p* = 0.286] or of the interaction cue condition x impulsivity group [χ^2^(2) = 0.974, *p* = 0.614]. However, there was a main effect of impulsivity group [χ^2^(1) = 5.196, *p* = 0.023], with the low impulsivity group having higher correlation values than the high impulsivity group across all cue conditions.

Further analysis of the correlation during the baseline period showed that there was no significant effect of the impulsivity group [χ^2^(1) = 2.594, *p* = 0.107] or sex [χ^2^(1) = 2.895, *p* = 0.089].

### Behavioral Performance

#### Number of Early Response Errors

**FIGURE 7:**
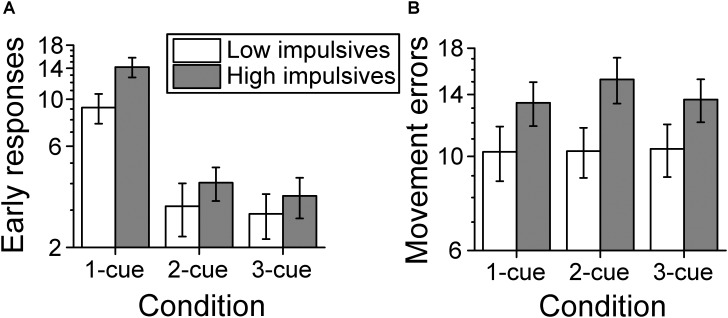
Behavioral performance. **(A)** Number of early response errors per cue condition and impulsivity group. The number of early errors decreased as number of cues increased and was greater for the high impulsivity group than for the low impulsivity group. **(B)** Number of movement errors per cue condition and impulsivity group. The number of movement errors was not affected by number of cues but was greater for the high impulsivity group than the low impulsivity group. The ordinates are in a log scale to be consistent with the log link used with the mixed model analysis. Error bars represent the standard error of the mean (Low impulsivity *N* = 24; High impulsivity *N* = 26).

Figure 7A shows the average number of early response errors per cue condition and impulsivity group. The analysis of the number of early response errors (also called errors of commission in the literature) indicated that there was a main effect of number of cues [χ^2^(2) = 159.661, *p* < 0.001; linear contrast: *z* = -10.695, *p* < 0.001], which indicates that the number of early response errors decreased as the number of cues increased. There was no significant effect of sex [χ^2^(1) = 1.538, p = 0.215]. However, we found a main effect of impulsivity group [χ^2^(1) = 4.903, *p* = 0.027], due to a greater number of early response errors for the high impulsivity group than for the low impulsivity group. There was no significant effect of the interaction number of cues condition x impulsivity group [χ^2^(2) = 1.239, *p* = 0.538].

#### Number of Movement Errors

Figure 7B shows the average number of movement errors per cue condition and impulsivity group. The analysis of the number of movement errors showed no significant effect of number of cues condition [χ^2^(2) = 1.597, *p* = 0.450], or of sex [χ^2^(1) = 1.713, *p* = 0.191]. However, there was a significant effect of impulsivity group [χ^2^(1) = 4.523, *p* = 0.033], due to a greater number of movement errors for the high impulsivity group than for the low impulsivity group. There was no significant effect of the interaction cue condition x impulsivity group [χ^2^(2) = 1.452, *p* = 0.484].

#### Reaction Time

The analysis of mean reaction time showed that there was a significant effect of number of cues [χ^2^(2) = 1017.618, *p* < 0.001; linear contrast: *z* = 31.177, *p* < 0.001], with reaction time increasing as the number of cues increased (mean RT (sem) for the 1, 2, and 3-cue condition was 372.6 ms (6.7 ms), 425.9 ms (6.2 ms), and 452.7 ms (6.5 ms), respectively). There was no significant effect of sex [χ^2^(1) = 0.173, *p* = 0.677], or of impulsivity group [χ^2^(1) = 0.004, *p* = 0.948], or of the interaction cue condition x impulsivity group [χ^2^(2) = 4.477, *p* = 0.107].

**FIGURE 8:**
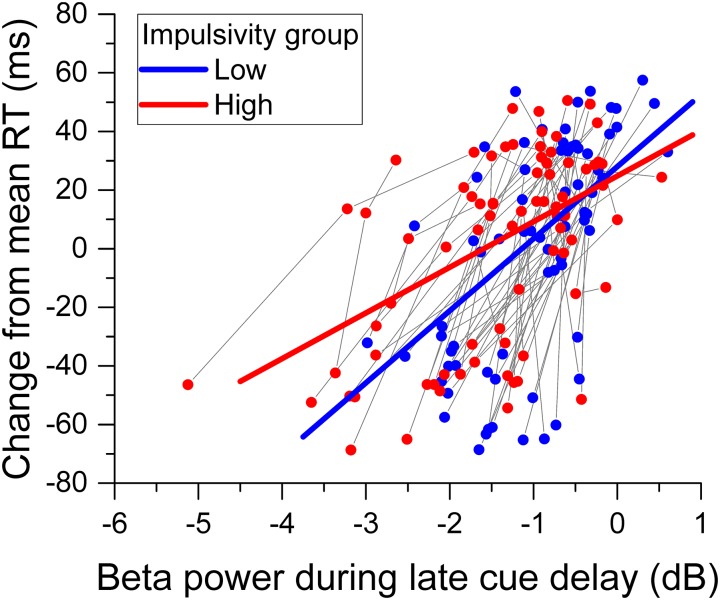
Relation between reaction time and beta band power. The scattergram shows the change of reaction time from the mean against beta desynchronization during the late cue period, for each participant and cue condition. The data from the same participant are connected by a gray line. The straight lines through the data points represent the best fit of the model. Data from the low impulsivity group are in blue, those from the high impulsivity group are in red.

The greater overall change of relative beta power during the cue delay period for the high impulsivity group than for the low impulsivity group with no concomitant significant difference in reaction time between groups raised the issue of how impulsivity affected the relation between reaction time and beta band power. The analysis of change in reaction time from the mean showed a significant main effect of beta desynchronization [χ^2^(1) = 77.380, *p* < 0.001], indicating that reaction time deflected positively from the mean with less beta power desynchronization. There was no significant main effect of impulsivity group [χ^2^(1) = 0.348, *p* = 0.555]. However, there was a significant relative beta power x impulsivity group interaction [χ^2^(1) = 5.213, *p* = 0.022]. Figure 8 shows the change in reaction time from the mean against relative beta power during the late cue period for each participant from the low and high impulsivity groups, as well as the regression lines from the model. The model shows that, for each group, reaction time deflected positively from the mean with smaller beta desynchronization during the late cue period. However, the slope was steeper for the low impulsives than for the high impulsives.

#### Movement Time

The analysis of mean movement time showed a significant effect of number of cues condition [χ^2^(2) = 14.086, *p* = 0.001]. The linear contrast [*z* = -3.706, *p* < 0.001] showed that movement time was shorter for greater number of cues (mean MT (sem) for the 1, 2, and 3-cue condition was 170.3 ms (6.5 ms), 167.0 ms (5.7 ms), and 162.8 ms (5.7 ms), respectively). However, there was no significant effect of sex [χ^2^(1) = 0.060, *p* = 0.807], impulsivity group [χ^2^(1) = 0.329, *p* = 0.566] or cue condition x impulsivity group interaction [χ^2^(2) = 4.977, *p* = 0.083].

## Discussion

We investigated whether the modulation of beta band oscillations associated with motor planning was differentiated by the level of the impulsivity personality trait. To this end we estimated the trait impulsivity of a cohort of healthy volunteers using the Barratt Impulsiveness Scale, and used magnetoencephalography to record their brain oscillatory activity while they performed an instructed-delay reaching task with different conditions of target uncertainty. We found that the decrease of beta band power during motor planning in a left fronto-central condition-related group of channels was associated with impulsivity. Specifically, the high impulsivity group had a greater decrease in beta power during the late cue period of the task than the low impulsivity group. In addition, we found that the beta band-mediated functional connectivity during the task between the posterior and left fronto-central condition-related groups of channels was also associated with impulsivity. In particular, the high impulsivity group had a weaker functional connectivity during the early cue period than the low impulsivity group. Furthermore, the two groups also diverged behaviorally, with high impulsives making more early response and movement errors in the task than low impulsives. We believe that these results shed new light on the neural mechanisms through which impulsivity affects action planning. Below we discuss the nature and implications of these findings in more detail.

### Effect of Impulsivity on Beta Band Power

We found that left fronto-central beta band power was modulated by the degree of uncertainty about the direction of the upcoming target (Figures 3B,C). Specifically, the decrease of beta band power was less pronounced as the number of cues, hence uncertainty, increased (Figures 3E,F). This is consistent with previous results (Praamstra et al., 2009; Tzagarakis et al., 2010, 2015; Rhodes et al., 2018). The effect of number of cues was visible early after cue onset (0–0.5 s; Figure 3E), and then intensified and encompassed more channels during the late cue period (0.5–1 s; Figure 3F). The results of the source analysis indicated that the decrease of beta band power was localized over sensorimotor cortical areas (Figure 4), which is also consistent with previous studies (Tzagarakis et al., 2010, 2015). Here, we show for the first time that beta power modulation associated with motor planning was affected by the degree of trait impulsivity, with high impulsivity leading to a greater beta power decrease than low impulsivity for all cue conditions at the late stage of movement planning. There was no effect of impulsivity on relative beta power during the early part of the task cue period or on beta power at baseline. Although we did not find an effect of impulsivity during the baseline period of the task, other studies have found an effect of impulsivity in resting state conditions (Lee et al., 2017; Threadgill and Gable, 2018). The different outcomes might be due to that the effect of impulsivity in resting state conditions, that is, in the absence of an upcoming action, may be different compared to its effect during a resting but anticipatory state in the context of a task. This needs to be explored further in future studies.

In addition to the effect of cue condition on the left fronto-central beta band power, we found a significant posterior modulation of beta band power (Figure 3A) during the early cue period that was negatively correlated with number of cues, that is, beta power decreased further as the number of cues increased (Figure 3D). The source analysis localized this effect to the occipito-parietal area, with a preponderance on the right (Figure 4). Although beta activity modulation is most often discussed in the context of motor processes, a decrease of beta power in parieto-occipital areas associated to visual stimulus onset or offset has been shown in other studies as well (Kloosterman et al., 2015; Meindertsma et al., 2017). Here, we found a tendency (not significant after Bonferroni correction) for a smaller change of beta power with number of cues in the high impulsivity group than in the low impulsivity group (Figure 3G). This result suggests that the perceptual-related modulation of beta power was less differentiated in the high impulsivity group than in the low impulsivity group, which may indicate a lesser contribution of visual updating on motor planning in the former than the latter. The results regarding functional connectivity (see below) provide additional indication of a different level of perceptuo-motor interaction between the two impulsivity groups.

### Effect of Impulsivity on Beta Band-Mediated Functional Connectivity

Given the presence of a posterior group and a left central group of channels (Figures 3A,B, 6A) with differential effect of number of cues on beta power during the early cue period, we examined their functional connectivity during that period of the task. To this end, we computed the trial by trial time-varying correlation of beta band amplitude between each pair of channels from the two groups. We found that there was a transient increase in the average correlation following cue onset (Figure 6B). This increase in correlation was more marked as the number of cues increased, possibly indicating the need for more perceptual input in motor planning when cues indicate more than one movement alternatives, and was weaker for the high impulsivity group than for the low impulsivity group across all cue conditions (Figure 6C).

Significant fronto-parietal connectivity in tasks with a motor component is well established (Gao et al., 2011). Impulsivity has been found to affect inter-area connectivity in more than one ways, depending on the task executed, and results have been at times conflicting (Vaidya and Gordon, 2013). Few studies have actually analyzed functional connectivity in the beta band, although Chen et al. (2008) reported low resting state beta connectivity in frontal MEG channels for patients with bipolar disorder. Resting state BOLD-based connectivity of dorsolateral premotor cortex (DLPMC) with dorsal attentional and executive control networks was found to be increased in low vs. high impulsivity, whereas the reverse was true for DLPMC connectivity with the default mode network (Shannon et al., 2011), suggesting a tendency for high impulsives to use less perceptual information when planning action than low impulsives. This is also consistent with other studies that have suggested that high impulsivity can be considered to be a state in which external information has less impact in the decision to act (Clark et al., 2006). This mechanism has also been suggested as the cause of differential behavioral performance and single unit activity in medial prefrontal cortex in an animal model of impulsivity (Donnelly et al., 2015).

It is unclear what the function of lower perceptual contribution to action planning is in the context of high impulsivity. One way to interpret these results would be that high impulsives follow the same strategy as low impulsives but are more likely to be cognitively/perceptually overwhelmed by the demands of the task. This would however lead to effects of impulsivity on RT (and possibly MT), as it would mean that high impulsivity would be associated with longer reaction times as cognitive load (i.e., number of cues) increases, which is not what was observed here (see also discussion below). It is more likely that, if the formation and quality of alternate movement plans result from the integration of various factors including perceptual updating, internal biases of the expectation of target location as well as stability of the internal representation of movement plans and ability to switch between them, high and low impulsives differ in the management of all these resources resulting in what can be called different strategies (Dickman and Meyer, 1988) for movement planning and decision making in general. This interpretation of the results also provides a possible connection between the planning and the decisional aspects of impulsivity (i.e., risk taking, delayed discounting) as the act of motor planning can be viewed as a choice process where high impulsivity introduces a certain amount of risk-taking even when the outcomes are equiprobable. Exploring the nature and neural basis of these aspects of high impulsivity planning should be the object of further study.

### Alpha Band

In a previous publication (Tzagarakis et al., 2015), we showed that occipito-parietal alpha band (8-12 Hz) power was also modulated by cue condition shortly after cue onset. However, there was no significant effect during the late cue period in contrast to beta power. That is the reason the present study focused on beta band activity, which is more directly relevant to motor planning than alpha band activity. Alpha band analysis (not shown) of the dataset used here, confirmed the early posterior alpha modulation by number of cues but showed no significant early or late main effect of or interaction with impulsivity. Therefore, although a role for alpha activity in the physiology of impulsivity cannot be excluded, and should be the object of further study, the role of beta oscillations in action planning and its modulation by impulsivity seems more critical.

### Effect of Impulsivity on Behavioral Measures

The results supported the hypothesis that high impulsivity would be associated with a greater number of early errors (Figure 7A). This was consistent with our previous findings in a larger cohort (Tzagarakis et al., 2013). The fact that the high impulsivity group had a greater decrease in relative beta power than the low impulsivity group suggests a causal connection with the increase in early response errors. First, it is well established that motor preparation is associated with a decrease of beta band power (Pfurtscheller and Lopes da Silva, 1999). In addition, it has been shown that movement execution is associated with further decrease of beta band power to a lower common level (Tzagarakis et al., 2010; Grent-’t-Jong et al., 2014; Heinrichs-Graham and Wilson, 2016). For that reason, the lower level of beta band power during action planning in high impulsives is closer to the level for movement execution, which presumably increased the probability of inadvertent early responses.

Despite these results, we found no effect of impulsivity on reaction (or movement) time in the task used. This is consistent with our previous findings (Tzagarakis et al., 2013), as well as other studies in the literature (e.g., Hagenhoff et al., 2013; Taylor et al., 2016), including a meta-analysis of studies of substance misuse which indicates that reaction time measures are not sensitive to these highly impulsive clinical populations, contrary to stop-signal times (Smith et al., 2014). Nevertheless, to further explore this issue, we performed a *post hoc* regression analysis using change in reaction time as the dependent variable, and beta desynchronization during the cue period, as well as impulsivity group as predictors. Rather than being a measure of overall task performance, change in reaction time allows exploring the way performance is managed across different task conditions. The analysis showed that, for successful trials, reaction time changed less with beta desynchronization for high impulsives than for low impulsives (Figure 8). In turn, this implies that although the higher beta desynchronization in high impulsivity increases the probability of an inappropriate response (early error), it leads to similar reaction time to low impulsives when responses are correct. This bolsters a view of high impulsivity as the manifestation of different, more error-prone strategies, rather than of a deficit in a specific cognitive mechanism (Dickman and Meyer, 1988).

Finally, we found that the high impulsivity group had more movement errors than the low impulsivity group independently from cue condition (Figure 7B). The task implementation imposed specific accuracy constraints for the movement trajectory and the time on target, thus requiring very accurate motor planning and execution. These results can therefore be interpreted as evidence for the role of the impulsivity trait not only in movement initiation and inhibition but also in defining an appropriate movement plan and then implementing it correctly. Determining the exact function of impulsivity in this sequence of neural events should be the object of further study.

### Movement Time

Movement time appeared to decrease as the number of cues increased, thus going in the opposite direction to reaction time. This effect was not linked to impulsivity. Higher perceptual load has been shown to decrease online correction in reaching tasks (Sandoval Similä and McIntosh, 2015) and could explain this result, considering that more cues correspond to a higher perceptual load.

### Sex Effect

We found no sex effect in any of the beta power and connectivity analyses as well as the behavioral analyses. Nevertheless, several, at times conflicting, studies, (e.g., Waldeck and Miller, 1997; Yuan et al., 2008) found sex effects on the manifestation of the impulsivity trait. This includes our own past work (Tzagarakis et al., 2013). Contrary to the present study however, that work involved an heterogenous and less well stratified dataset and also lacked the benefit of neural data which we have used here to define the most appropriate analyses. We believe that further exploration of the sex effects associated with impulsivity is needed to clarify the interaction between sex and impulsivity.

### Pharmacological Implications

The findings of this study indicate that the decrease of beta band power associated with motor planning was greater, and the beta band-mediated perceptuomotor connectivity was weaker in the high impulsivity group than the low impulsivity group. Consequently, the monitoring of beta band activity could potentially provide markers of efficacy for pharmacological agents used to decrease impulsivity in clinical settings.

For example, the core neurophysiological marker of Parkinson’s disease is the pathological increase of beta band activity in motor cortico-basal ganglia loops which is associated with a difficulty in initiating movements (Brittain et al., 2014). The use of dopamine agonists for the treatment of Parkinson’s disease decreases beta band activity and improves motor symptoms, but is also known to increase impulsivity (Stenberg, 2016). Conversely, the use of medication that enhances dopamine release (amphetamines) is known to decrease impulsive behavior in ADHD (De Crescenzo et al., 2017) while dopamine antagonists have the same effect in other conditions (e.g., Van den Eynde et al., 2008). The role of dopamine in regulating impulsive behavior as well as the definition of the optimal clinical use of dopaminergic agents for modulating it in various conditions thus merits continued exploration.

Furthermore, lithium treatment which has a well-known anti-impulsivity and anti-suicidality effect (Hollander et al., 2005; Hayes et al., 2016; Smith and Cipriani, 2017), and improves decision making in bipolar disorder (Adida et al., 2015) increases beta band activity at rest (Thau et al., 1989) and, even more consistently, during task performance (Atagün, 2016). Given the lack of a complete understanding of the mechanism of action of lithium (Malhi and Outhred, 2016), further study of its electrophysiology in the context of impulsivity would provide much needed insight into both the nature of its therapeutic effects as well as the impulsivity trait itself. Our prediction based on the findings presented here is that lithium increases pre-action relative beta power, thus making impulsive actions less likely and increases fronto-parieto-occipital connectivity thus allowing for more influence of perceptual input on choice.

### Limitations

The lack of structural MRI data did not allow extracting high-resolution anatomical information from this dataset and limited the main analyses to channel space. However, the head shape data recorded permitted the fitting of a source model allowing the general localization of beta modulation associated with the task. The sensorimotor areas thus identified are in concordance with previous results from datasets with higher quality source analyses (Tzagarakis et al., 2010, 2015). Nevertheless, future studies should allow for high resolution source space analysis of the effects of impulsivity on beta activity.

The use of a two-step analysis, that is, using the dependent samples correlation between beta band power and number of cues to select groups of channels that were then further analyzed for impulsivity could be viewed as a potential source of bias due to “double dipping” (Kriegeskorte et al., 2009). However, the effect of impulsivity was not part of the channel selection process and therefore was unaffected by such potential bias.

The analysis of functional connectivity in channel space poses the risk of type I error due to volume conduction effect. This means that the correlation between groups of channels may occur because of contamination from a common source (Cohen, 2014; Garcés et al., 2016). However, here the two channel groups exhibited a reverse effect of cue condition on beta power which is not what would be expected with a volume conduction effect. In addition, there is no reason to expect systematic differences in volume conduction that could explain the differences in functional connectivity between impulsivity groups. In any case, further study of inter-area beta connectivity associated with impulsivity is warranted.

We selected the Barratt Impulsiveness Scale questionnaire to evaluate trait impulsivity in this study because of its wide use in both health and disease. This measure represents however only one aspect of this multi-faceted construct. Different trait impulsivity measures as well as different tasks, can highlight different aspects of impulsivity. For example, an fMRI study, with a similar size dataset to the one used in this study, using a different motor task more heavily weighted toward late response inhibition and a different self-report scale (UPPS-P Impulsive Behavior Scale; Whiteside et al., 2005) found no significant neural effect of impulsivity on the motor or occipito-parietal cortex but rather on the inferior frontal gyrus, while there was no correlation with the BIS scale (Wilbertz et al., 2014).

Movement inhibition and termination is associated with an increase in beta power known as post-movement beta rebound (PMBR; Heinrichs-Graham et al., 2017), which has been shown to be modulated by physiological (e.g., Gaetz et al., 2010) as well as pathological (e.g., Vakhtin et al., 2015) factors. Although the focus of the present work has been the effect of impulsivity on beta oscillations during movement planning, possible effects further downstream in the motor sequence, such as on PMBR, need to be the elucidated in the future.

It would be desirable to test the effect of impulsivity with a finer grain than what was done in the present study. However, dividing the participants in more subgroups of fewer participants reduces the confidence of the estimates (i.e., increases the standard error) of the neural data within each subgroup. Using the Barratt scores to dichotomize this moderately-sized dataset into high and low impulsivity groups proved to be the most fruitful approach with this dataset. Larger datasets with stratified recruitment should allow for finer-grained correlational analysis of self-reported impulsivity. Larger cohorts, result replication and multiple analytical approaches will help confirm the key neurophysiological effects associated with impulsivity.

Although this study focused on impulsivity in healthy individuals, we thought it useful to discuss potential implications of the results for some clinical conditions. However, further validation in clinical populations is warranted to generalize our conclusions. It is important nevertheless to note that the range of high impulsivity scores seen in our sample is comparable to the range of scores seen in a variety of clinical populations (e.g., Herbort et al., 2016; Sher et al., 2016; Aguglia et al., 2018). This should come as no surprise since high impulsivity by itself is not a disease and high values of the impulsivity trait (especially when measured with the BIS which does not ask clinical symptom-related questions), can be present in both healthy individuals and patients affected by one of the conditions associated with high impulsivity. What makes the impulsivity trait important in disease is its interaction with other disease features, such as mood instability and altered cognition. Such interaction can be at the source of significant morbidity and make the impulsivity trait in disease a reasonable therapeutic target.

## Conclusion

In summary, we analyzed beta oscillatory activity in healthy volunteers with high and low impulsivity during an instructed-delay motor task. High impulsivity amplified the decrease in beta band power associated with motor planning, and reduced beta band functional connectivity between posterior and left fronto-central areas. The greater reduction of beta band activity in high impulsives brings the level of beta band power during motor planning closer to its level during the movement which may explain their increased number of early response errors. These findings support the hypothesis of the important role of beta oscillations on the effect of impulsivity during goal directed actions and decisions. Therefore, these results open the way for further study of the role of beta band activity in impulsivity, especially in the context of disease and therapeutics.

## Author Contributions

CT and RR planned the experiments. CT recorded the data. CT, AT, and GP analyzed the data. CT and GP wrote the article.

## Conflict of Interest Statement

The authors declare that the research was conducted in the absence of any commercial or financial relationships that could be construed as a potential conflict of interest.
